# The Enigmatic Role of Lipids in Cilia Signaling

**DOI:** 10.3389/fcell.2020.00777

**Published:** 2020-08-11

**Authors:** Inna V. Nechipurenko

**Affiliations:** Department of Biology and Biotechnology, Worcester Polytechnic Institute, Worcester, MA, United States

**Keywords:** cilia, flagella, lipids, polyphosphoinositides, cholesterol

## Abstract

Primary cilia are specialized cellular structures that project from the surface of most cell types in metazoans and mediate transduction of major signaling pathways. The ciliary membrane is contiguous with the plasma membrane, yet it exhibits distinct protein and lipid composition, which is essential for ciliary function. Diffusion barriers at the base of a cilium are responsible for establishing unique molecular composition of this organelle. Although considerable progress has been made in identifying mechanisms of ciliary protein trafficking in and out of cilia, it remains largely unknown how the distinct lipid identity of the ciliary membrane is achieved. In this mini review, I summarize recent developments in characterizing lipid composition and organization of the ciliary membrane and discuss the emerging roles of lipids in modulating activity of ciliary signaling components including ion channels and G protein-coupled receptors.

## Introduction

Cilia (or flagella) are hair-like cellular projections that are highly conserved across eukaryotes (Carvalho-Santos et al., 2011). Based on their structural features, cilia are classified into motile and non-motile subtypes. Non-motile cilia, also known as primary cilia, are present on nearly all vertebrate cell types and function as signaling hubs during development and in differentiated tissues. In fact, components of all major signaling pathways including Hedgehog (Hh), Wnt, Notch, transforming growth factor β, G protein-coupled receptors (GPCRs), receptor tyrosine kinases, and extracellular matrix receptors localize to cilia and require these organelles for proper transduction (Mykytyn and Askwith, 2017; Anvarian et al., 2019). While most cells in the human body possess a solitary primary cilium, motile cilia are also present on the surface of some specialized cells in the airway, oviduct, and brain ventricles (Brooks and Wallingford, 2014). Like their non-motile counterparts, motile cilia can detect and transmit diverse sensory cues in addition to beating and propelling fluids (Bloodgood, 2010). Due to the central role of cilia in signaling and their nearly ubiquitous distribution across human tissues, perturbations in cilia structure and/or function manifest in a spectrum of genetic disorders called ciliopathies (Reiter and Leroux, 2017). These diseases affect most human organ systems and present with pleiotropic developmental and adult phenotypes that include blindness, kidney and heart disease, obesity, and cognitive deficits (Badano et al., 2006).

Since the discovery of motile cilia in the 17th century by Antonie van Leeuwenhoek until the early 2000s, cilia research was rather scarce and focused primarily on the axoneme – the microtubule backbone of the organelle (Bloodgood, 2009). It was at the dawn of the 21st century, when the sensory functions and clinical relevance of cilia were broadly demonstrated, that an interest in cilia surged, and attention of the scientific community shifted to the ciliary membrane. Unlike other cellular organelles, cilia are not fully enclosed by membrane. Instead, the ciliary membrane is continuous with the plasma membrane, and at their base, cilia are exposed to the cytosol. Despite continuity with the plasma membrane, the ciliary membrane exhibits a unique protein and lipid composition that is maintained, at least in part, by multiple diffusion barriers at the cilia base (Verhey and Yang, 2016). During the last two decades, much progress has been made in identifying the protein constituents of the ciliary membrane and molecular mechanisms of their trafficking in and out of cilia (Nachury and Mick, 2019). In contrast, the ciliary lipidome or mechanisms controlling its establishment are only starting to come to light.

This mini-review briefly summarizes current knowledge about ciliary membrane lipid composition and the molecular mechanisms that regulate ciliary lipid content. I also discuss the emerging roles of lipids in cilia signaling and outline major outstanding questions regarding the roles of lipids in modulating cilia-based pathways and shaping ciliary membrane morphology. Addressing these questions in the future may provide insight into human pathological conditions linked to altered membrane lipid constitution.

## Cilia Architecture

**FIGURE 1:**
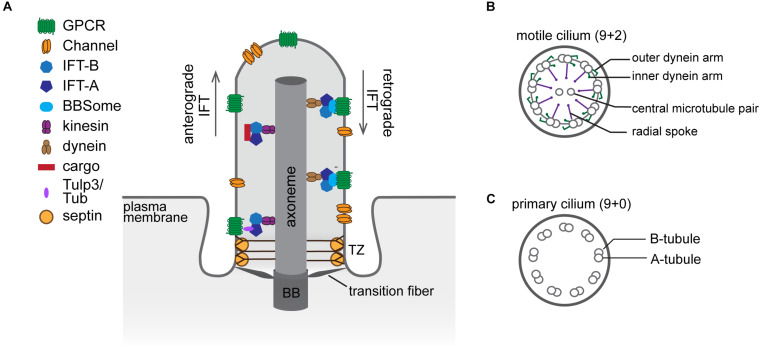
The structural organization of the cilium. **(A)** Diagram of a cilium depicting major structural components and ciliary sub-compartments. **(B,C)** Cross-section schematics of a typical motile **(B)** and primary **(C)** cilium. BB, basal body; TZ, transition zone.

The cilium is comprised of a core microtubule-based structure called the axoneme ensheathed by a specialized membrane. Nine radially symmetric microtubule doublets (A- and B-tubules) of the axoneme extend from the basal body – a modified mother centriole, which nucleates the axoneme and anchors the cilium at the cell surface (Ishikawa and Marshall, 2011; Figure 1A). In addition to the nine peripheral microtubule doublets, the axoneme of the motile cilium typically contains a central pair of singlet microtubules required for ciliary beating (9 + 2 arrangement), while the axoneme of the primary cilium lacks it (9 + 0 arrangement) (Satir and Christensen, 2007; Figures 1B,C). Most motile cilia also have radial spokes and inner and outer dynein arms attached to the microtubule doublets of the axoneme to drive motility (Ishikawa, 2017; Figure 1B).

Since there are no ribosomes inside the cilium, all ciliary proteins are imported from the cytosol. The transition zone (TZ), which constitutes the proximal 0.5–1.0 μm of the axoneme, is comprised of several macromolecular complexes that serve as a gate controlling selective entry and exit of ciliary cargoes. At the ultrastructural level, the TZ is characterized by Y-shaped fibers (Y-links) connecting the microtubule doublets of the axoneme to the ciliary membrane (Blacque and Sanders, 2014; Garcia-Gonzalo and Reiter, 2017; Figure 1A). Together with the transition fibers, which anchor the basal body to the membrane, Y-links provide a physical barrier that separates the cilium proper from the cytoplasm, and the membrane attachment points of the transition fibers demarcate the boundary between the plasma and ciliary membranes. Cilia assembly and maintenance are mediated by a bi-directional transport system called intraflagellar transport (IFT). Microtubule motors in conjunction with three multi-subunit complexes – IFT-A, IFT-B, and the Bardet–Biedl syndrome (BBS)ome – traffic proteins along the axoneme between the ciliary base and tip (reviewed in Taschner and Lorentzen, 2016; Wingfield et al., 2018; Figure 1A). Notably, mutations in genes encoding components of the basal body, TZ, and IFT are associated with ciliopathies including Meckel-Gruber and Joubert syndromes, nephronophthisis, polycystic kidney disease, and Bardet–Biedl syndrome, underscoring the importance of cilia in human health (Reiter and Leroux, 2017).

## Lipid Composition of the Ciliary Membrane

### Polyphosphoinositide Distribution and Roles in Ciliary Protein Trafficking

**FIGURE 2:**
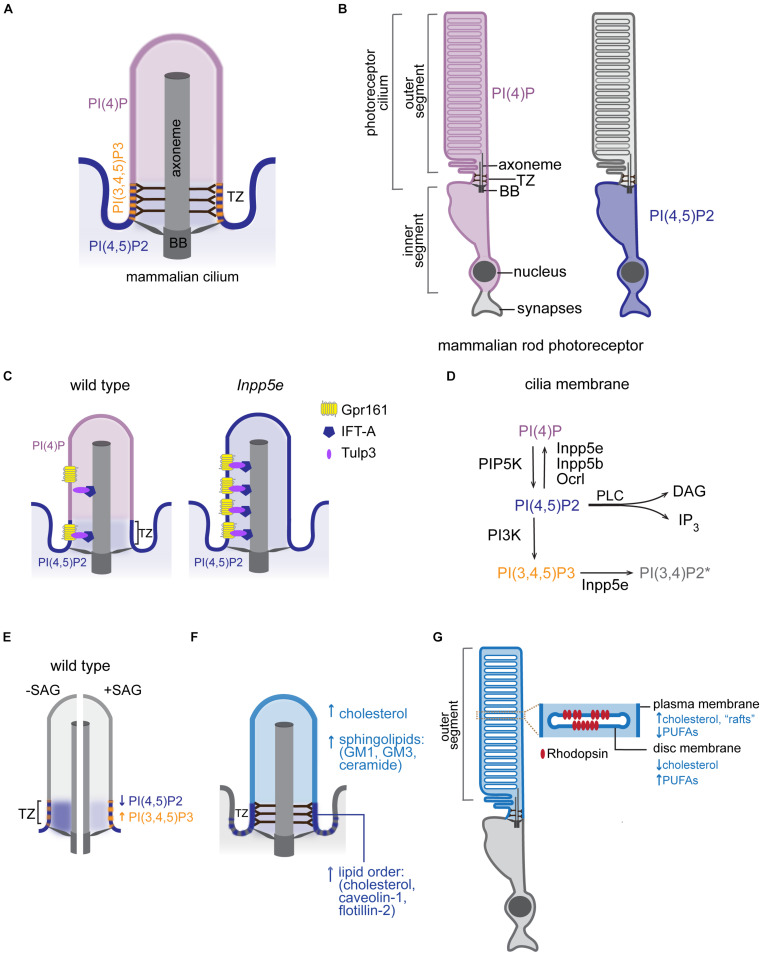
Lipid composition of the ciliary membrane. **(A)** Schematic representation of PPI distribution in the ciliary membrane of mammalian cells. BB, basal body; TZ, transition zone. **(B)** Distribution of the indicated PPI species in rod photoreceptors of mammalian retina. **(C)** Intraciliary localization of PI(4)P and PI(4,5)P2 in mammalian wild-type and *Inpp5e* mutant cells. Distribution of Tulp3/IFT-A trafficking complex and its GPCR cargo in control and *Inpp5e* mutant cells is also shown. **(D)** PPI species and a subset of PPI metabolizing enzymes that have been reported inside cilia. The presence of PI(3,4)P2 in the ciliary membrane is inferred based on intraciliary localization of Inpp5e and its substrate PI(3,4,5)P3 (Moore et al., 2016; Dyson et al., 2017). DAG, diacylglycerol, IP_3_, inositol 1,4,5-trisphosphate, PLC, phospholipase C. **(E)** Diagrammatic representation of changes in PPI composition at the TZ in response to Hh pathway activation. Blue and orange arrows mark direction of the observed changes in PI(4,5)P2 and PI(3,4,5)P3 levels, respectively, in the wild-type SAG-treated or untreated control cells. **(F)** A schematic representation of major raft-associated components (proteins and lipids) known to be enriched in the ciliary membrane. Dashed lines at cilia base represent condensed lipid microdomains detected by Laurdan microscopy in some cell types. **(G)** A schematic diagram of the mammalian rod photoreceptor. Inset shows an enlarged view of the disc and surrounding OS plasma membrane highlighting their distinct lipid content. PUFAs, polyunsaturated fatty acids.

Considerable progress has been made in understanding how cilia establish their unique protein content (reviewed in Garcia-Gonzalo and Reiter, 2017; Mukhopadhyay et al., 2017; Morthorst et al., 2018; Nachury and Mick, 2019). On the other hand, much remains to be discovered about how cells maintain the ciliary membrane lipid identity. Some lipid biosynthetic enzymes localize to distinct sub-ciliary compartments and locally modulate membrane lipid composition. Conversion of polyphosphoinositides (PPIs) by multiple kinases and phosphatases provides the best-known example of lipid generation at local sites in the ciliary membrane. Polyphosphoinositides are signaling lipids generated by reversible phosphorylation of phosphatidylinositol (PI) at positions 3, 4, and 5 of its inositol ring (Balla, 2013). These phosphorylation derivatives of PI populate distinct membrane domains within cells, where they regulate many aspects of cellular physiology (Di Paolo and De Camilli, 2006). The ciliary membrane in mammals and sea urchin contains high levels of phosphatidylinositol-4-phosphate [PI(4)P] relative to the adjacent plasma membrane (Chávez et al., 2015; Garcia-Gonzalo et al., 2015). In contrast, phosphatidylinositol-4,5-bisphosphate [PI(4,5)P2] is largely depleted from the ciliary membrane in mammals, *Caenorhabditis elegans*, *Drosophila melanogaster*, and *Trypanosoma brucei*. Instead, PI(4,5)P2 localizes to distinct membrane domains at the cilia base creating a sharp boundary in PPI composition (Chávez et al., 2015; Garcia-Gonzalo et al., 2015; Jensen et al., 2015; Park et al., 2015; DiTirro et al., 2019; Dyson et al., 2017; Figure 2A). In retinal pigmented epithelial cells and primary mouse embryonic fibroblasts, PI(4,5)P2 is concentrated at the TZ, which also contains phosphatidylinositol-3,4,5-trisphosphate [PI(3,4,5)P3] (Dyson et al., 2017). Conversely, in *C. elegan*s neurons and *T. brucei*, PI(4,5)P2 is enriched in an endocytic membrane domain (periciliary membrane compartment/ciliary pocket), which lies proximal to the TZ (Demmel et al., 2014; Jensen et al., 2015; DiTirro et al., 2019). In photoreceptors, while PI(4,5)P2 is also largely excluded from the outer segment (OS), which is a modified cilium, PI(4)P localizes to the OS as well as inner segment and perinuclear regions (Nasuhoglu et al., 2002; Finkelstein et al., 2020; Figure 2B). More studies are needed to systematically evaluate PPI composition across all ciliated cell types and to better understand the physiological significance of cell-specific differences in PPI composition and sub-ciliary distribution.

How does the cilium maintain a unique PPI distribution? Inpp5e inositol polyphosphate-5-phosphatase, which converts PI(3,4,5)P3 and PI(4,5)P2 into PI(3,4)P2 and PI(4)P, respectively, localizes to mammalian cilia (Bielas et al., 2009; Jacoby et al., 2009; Luo et al., 2012, 2013). Mutations in *INPP5E* cause Joubert and MORM (mental retardation, truncal obesity, retinal dystrophy, and micropenis) syndromes in humans, and *Inpp5e* knockout mice display phenotypes consistent with ciliopathies (Jacoby et al., 2009). In the absence of *Inpp5e*, PI(4,5)P2 accumulates in the cilium, while ciliary PI(4)P levels drop (Chávez et al., 2015; Garcia-Gonzalo et al., 2015; Figure 2C). Similarly, in fly and worm sensory neurons, loss of INPP5E orthologs leads to increased PI(4,5)P2 levels in cilia (Park et al., 2015; DiTirro et al., 2019). Together, these findings are consistent with the model that PI(4,5)P2 diffuses laterally from the plasma to the ciliary membrane, where it is converted to PI(4)P by Inpp5e. Two other inositol polyphosphate-5-phosphatases (Inpp5b and Ocrl) have been reported to localize inside cilia of mammalian cells (Bielas et al., 2009; Jacoby et al., 2009; Luo et al., 2012, 2013). Mutations in human *OCRL* cause Lowe syndrome, a multisystemic disorder with characteristics of a ciliopathy, and cilia from Lowe syndrome patient fibroblasts contain high levels of PI(4,5)P2 and low levels of PI(4)P similarly to *Inpp5e* mutant cilia (Coon et al., 2012; Prosseda et al., 2017). Therefore, it is likely that several inositol polyphosphate-5-phosphatases contribute to the ciliary membrane PPI composition (Figure 2D). It is tempting to speculate that differences in cell and tissue distribution of PPIs and their metabolizing enzymes might contribute to symptomatic variability observed in patients carrying mutations in inositol polyphosphate-5-phosphatases (e.g., cataracts in Lowe and MORM patients versus retinitis pigmentosa in Joubert patients) (Hampshire et al., 2006; Madhivanan et al., 2012; Wang et al., 2018).

Beside inositol polyphosphate-5-phosphatases, several other phospholipid-metabolizing enzymes have been reported in the photoreceptor OS, where lipid metabolism has been extensively studied (reviewed in Giusto et al., 2000; Rajala, 2020; Wensel, 2020). Among these enzymes are phosphatidylinositol 3-kinase, which converts PI, PI(4)P, and PI(4,5)P2 into PI(3)P, PI(3,4)P2, and PI(3,4,5)P3, respectively, and phospholipase C, which cleaves PI(4,5)P2 to generate second messengers inositol 1,4,5-trisphosphate and diacylglycerol (Figure 2D). More studies are needed, however, to fully understand how ciliary phospholipid composition is modulated by these enzymes in different contexts, and how it contributes to cilia-mediated cellular functions. Since PPIs constitute <1% of total phospholipid mass in eukaryotic cells, with PI(4)P and PI(4,5)P2 being most abundant (∼0.05% each) (Fliesler and Anderson, 1983; Lemmon, 2008), PPI detectability in ciliary membranes presents a technical challenge. Development of more sensitive tools such as a recently reported ELISA-based method (He et al., 2016) is necessary to accurately measure these low-abundance lipids.

Although it remains to be determined whether diffusion barriers at the cilia base directly influence PPI distribution, the intact TZ is required for ciliary localization of Inpp5e. Mutations in TZ genes *Tctn1*, *Tmem231*, *B9d1*, and *Mks1* disrupt Inpp5e ciliary localization (Garcia-Gonzalo et al., 2015; Roberson et al., 2015; Slaats et al., 2016; Goetz et al., 2017). The same genes are also necessary for ciliary localization of a small GTPase Arl13b, which regulates trafficking of several ciliary proteins, including Inpp5e. Thus, it is conceivable that the Tmem231/B9d1/Mks1/Tctn1 TZ complex localizes Inpp5e to the cilium via Arl13b, thereby indirectly regulating ciliary PPI distribution (Garcia-Gonzalo et al., 2011; Humbert et al., 2012).

PPIs can also directly bind to transmembrane proteins (Balla, 2013). Interestingly, the TZ levels of Mks1/Tctn1/Tmem231/B9d1 following SAG (Smoothened receptor agonist) treatment are lower in *Inpp5e* null compared to wild-type embryonic fibroblasts. Additionally, cilia base localization of the oligomeric GTPase Septin2 was similarly reduced under these conditions (Dyson et al., 2017). Septins interact with phospholipids including PI(4,5)P2, which in turn facilitate septin filament polymerization (Mostowy and Cossart, 2012). Like TZ proteins, septins localize to the cilia base, where they are proposed to form a diffusion barrier between the plasma and ciliary membranes and regulate localization of select TZ proteins including Tmem231 and B9d1 (Hu et al., 2010; Chih et al., 2012). Catalytic activity of Inpp5e is required for proper localization of TZ proteins and Septin2; therefore, it is likely that Inpp5e-modulated PPI composition at the cilia base can dynamically regulate TZ assembly. Future studies are needed to determine whether PPIs regulate TZ composition via direct binding to TZ proteins, indirectly by controlling Septin2 localization, or through other mechanisms.

In addition to regulating the TZ, PPIs play a key role in ciliary import of channels and GPCRs (Badgandi et al., 2017). The tubby family proteins TUB and TULP3 bind membrane PI(4,5)P2 and IFT-A and thereby serve as adaptors for delivery of transmembrane proteins into the cilium (Mukhopadhyay et al., 2010). The current model posits that the interaction of TUB/TULP3 with PI(4,5)P2 in the plasma membrane facilitates association of TUB/TULP3 with transmembrane proteins that are subsequently transported into the cilium via the IFT-A complex. Since the TUB/TULP3 interaction with protein cargoes is PI(4,5)P2-dependent, absence of PI(4,5)P2 in the ciliary membrane causes TUB/TULP3 cargoes to be released inside the cilium after traversing the TZ (Badgandi et al., 2017). Consistent with this model, depletion of Inpp5e and subsequent intraciliary accumulation of PI(4,5)P2 results in increased levels of Tulp3/IFT-A proteins and their transmembrane cargoes such as GPCR Gpr161 – a negative regulator of Shh signaling – inside cilia (Mukhopadhyay et al., 2013; Chávez et al., 2015; Garcia-Gonzalo et al., 2015; Figure 2C). Other ciliary proteins including components of the BBSome (e.g., BBS5) and the exocyst can bind PPIs *in vitro* suggesting a broad role for phospholipids in mediating ciliary protein trafficking (Liu et al., 2007; Nachury et al., 2007; Jin et al., 2010). Notably, recent cryo-electron microscopy structures of the native BBSome from bovine retina suggested that BBS5 may not bind PPIs *in vivo* or may do so via an unknown motif or after a conformational change (Singh et al., 2020).

Recent studies in mammals and *C. elegans* demonstrated that, similar to ciliary protein composition, PPI content of the ciliary membrane is dynamic and can change in response to signaling. For example, *C. elegans* mutants in *odr-1*, which encodes a receptor guanylyl cyclase, display elevated levels of intraciliary PI(4,5)P2 relative to wild type in a specialized sensory neuron type (DiTirro et al., 2019). In mammals, activation of Hh signaling with SAG increases PI(3,4,5)P3 while decreasing PI(4,5)P2 levels at the TZ (Dyson et al., 2017; Figure 2E). The latter study also showed that TZ levels of both PPI species were higher in *Inpp5e* null compared to wild-type cells upon SAG treatment, suggesting that Inpp5e is responsible, at least in part, for signaling-dependent modulation of PPI composition at the TZ. In the rod OS, several studies reported activation of PI-metabolizing enzymes in response to light as well as light-dependent changes in PI(4)P and PI(4,5)P2 levels (reviewed in (Giusto et al., 2000; Wensel, 2020). However, the direction of change in PPI composition differed among studies, and the physiological significance of these effects requires further investigation. It will be interesting to examine whether levels of other ciliary lipids are also modulated by signaling across cell types.

### Microdomains of High Lipid Order

Early studies in diverse biological systems detected high levels of sterols and sphingolipids in the ciliary membrane, suggesting the presence of ordered lipid domains (i.e., “lipid rafts”) (Montesano, 1979; Souto-Padron and De Souza, 1983; Kaneshiro et al., 1984; Chailley and Boisvieux-Ulrich, 1985). More recently, sphingolipids including ceramide and raft-associated gangliosides GM1 and GM3 have been identified in primary cilia of Madin–Darby Canine Kidney (MDCK) epithelial cells by immunofluorescence (Janich and Corbeil, 2007; He et al., 2012; Figure 2F). Sphingolipids have also been detected in pure intact flagella of *T. brucei* using reverse-phase liquid chromatography high resolution tandem mass spectrometry (Serricchio et al., 2015). Membrane microdomains enriched in cholesterol and sphingolipids are resistant to detergent solubilization, and detergent-resistant membranes have been used as a proxy for rafts in studies probing lipid-raft composition (Farnoud et al., 2015). Caveolin-1 – an intra-membranous protein that stabilizes cholesterol-rich raft domains – localizes to the TZ in a cholesterol-dependent manner in mammalian cells and is present in the detergent resistant membranes of the photoreceptor OS (Nair et al., 2002; Lajoie et al., 2009; Schou et al., 2017). Similarly, another lipid raft scaffold flotillin-2 was detected at the TZ in epithelial cells (Schou et al., 2017; Figure 2F). In further support of the raft-like composition of ciliary membranes, the TZ membrane in *Chlamydomonas reinhardtii* is exceptionally resistant to detergent extraction, and Laurdan microscopy of *T. brucei* and MDCK cells showed condensed lipid microdomains in the trypanosome flagella and at the base of primary cilia (Kamiya and Witman, 1984; Vieira et al., 2006; Tyler et al., 2009). Collectively, these studies suggest that the ciliary membrane has unique lipid composition with distinct membrane microdomains. More research is needed, however, to determine how distinct membrane lipid domains form and contribute to cilia function.

While select phosphoinositide-metabolizing enzymes localize to cilia and directly modify intraciliary PPI content, none of the enzymes involved in sphingolipid or cholesterol metabolism have been identified inside the cilium to date. The “picket fence model” of membrane compartmentalization may provide one possible mechanism for ciliary lipid organization. This model posits that transmembrane proteins anchored to the actin network act as a “picket fence” impeding diffusion of the adjacent lipid molecules via steric hinderance and hydrodynamic slowing effects (Kusumi et al., 2012). In fact, entire raft assemblies can be confined to distinct membrane compartments by the “picket fence” according to this model. Many ciliary proteins are transmembrane, and therefore may form “pickets” to restrict diffusion of membrane molecules. Furthermore, using cryo-electron tomography, a recent study demonstrated that actin filaments surround and are intertwined with microtubules of the axoneme inside the cilia of MDCKII cells, adding further credence to the “picket fence” model as a possible mechanism of the ciliary membrane compartmentalization (Kiesel et al., 2020). Future work will need to experimentally test this model of ciliary membrane organization and determine whether same or different mechanisms regulate compartmentalization of ciliary membranes across cell types.

## Lipids in Cilia-Based Signaling

### PPI-Dependent Transmembrane Signaling

PPIs are key mediators of cell signaling in eukaryotes. At the plasma membrane, phospholipase C-dependent hydrolysis of PI(4,5)P2 downstream of growth factor receptors and GPCRs generates second messengers that amplify and transmit signaling from the cell surface downstream (Falkenburger et al., 2010). Furthermore, PI(4,5)P2 and PI(3,4,5)P3 facilitate assembly of signalosomes by recruiting different classes of proteins with lipid-binding domains (reviewed in Prestwich, 2004; Rajala, 2010; Hammond and Burke, 2020). Among PI(3,4,5)P3 interacting proteins are guanine nucleotide exchange factors and GTPase activating proteins for small GTPases as well as kinases and signaling scaffold proteins (Balla, 2013). In photoreceptor cilia, light stimulates PI(3,4,5)P3 binding and subsequent activation of the kinase Akt1 - a major signaling protein downstream of receptor tyrosine kinases (Li et al., 2008). Growth factor-dependent activation of Akt has also been reported at the cilia base in other cellular contexts (Zhu et al., 2009; Wang et al., 2015; Suizu et al., 2016; Walia et al., 2019). More studies are needed, however, to address the contribution of PPIs and their metabolites to cilia-based signaling in different cellular contexts.

### Lipid-Dependent Regulation of Ion Channels

Membrane lipids, including phospholipids and cholesterol, can also directly modulate ion channels. For example, PI(4,5)P2 binds to and regulates the activity of voltage- and ligand-gated ion channels, inward rectifier channels, and transporters (reviewed in Suh and Hille, 2008; Duncan et al., 2020). Transient receptor potential (TRP) channels (e.g., PKD2, TRPM4, and TRPC1), voltage-gated potassium channels, cyclic nucleotide-gated channels, and epithelial sodium channels are all targets of PI(4,5)P2-dependent modulation and localize to cilia (Womack et al., 2000; Raychowdhury et al., 2005; Suh and Hille, 2008; Enuka et al., 2012; Flannery et al., 2015; Sanchez et al., 2016). The ciliary channels TRPM4 and PKD2, the latter of which is mutated in autosomal dominant polycystic kidney disease, can also bind cholesterol, suggesting that both lipids may regulate these channels’ activity (Autzen et al., 2018; Wang et al., 2019). Another ciliary channel TRPV4 possesses cholesterol recognition motifs, and both TRPV4 and TRPC1 depend on caveolin-1 and cholesterol for proper positioning in the plasma membrane (Bergdahl et al., 2003; Brazer et al., 2003; Gradilone et al., 2007; Kumari et al., 2015). Since both caveolin-1 and cholesterol have been detected in the ciliary membrane, it is possible that similar mechanisms contribute to TRPV4 and TRPC1 ciliary localization. Function of olfactory cyclic nucleotide-gated channels is also altered by cholesterol depletion (Brady et al., 2004), and both olfactory and cone cyclic nucleotide-gated channels are inhibited by PI(3,4,5)P3 (Zhainazarov et al., 2004; Brady et al., 2006; Bright et al., 2007). Taken together, these studies suggest that PPI and cholesterol compartmentalization of the ciliary membrane may be of major significance for proper function of cilia-localized ion channels. In *C. elegans*, polyunsaturated fatty acids also modulate function of TRPV ciliary channels, although it remains to be tested whether they do so via direct interactions (Kahn-Kirby et al., 2004). More work is needed to address the contribution of specific lipids to localization and function of different ciliary channels.

### Lipid-Mediated Regulation of GPCRs

In addition to regulating channels, membrane lipids interact with and modulate multiple aspects of protein receptor physiology including oligomerization and signaling dynamics. For example, PI(4,5)P2 can bind and stabilize the active conformation of several class A GPCRs (Yen et al., 2018). Many class A GPCRs are present in cilia, where they may be similarly regulated by PPIs (Anvarian et al., 2019). Some ciliary GPCRs transiently pool in the “intermediate compartment” demarcated by the TZ distally and the transition fibers proximally before exiting or re-entering the cilium. This region is enriched in PI(4,5)P2 and may function as a distinct GPCR signaling domain (Ye et al., 2018).

The prototypical GPCR rhodopsin is enriched in the disc membrane of the photoreceptor OS. The rod OS contains a stack of closed membranous compartments (discs) encased by the OS plasma membrane (Figure 2G). Although discs form by evagination of the plasma membrane at the base of the OS followed by apical displacement, disc and OS membrane display distinct lipid composition (Boesze-Battaglia et al., 1994; Ding et al., 2015). For example, the disc membrane is enriched in polyunsaturated fatty acids and low in cholesterol relative to the surrounding OS membrane, suggesting an elaborate lipid sorting mechanism at the base of photoreceptor cilia (Aveldano and Bazan, 1983; Boesze-Battaglia and Schimmel, 1997; Nair et al., 2002; Figure 2G). Unique lipid content of disc and OS plasma membrane is critical for photoreceptor function, as aberrant distribution of cholesterol in the OS membranes is associated with photoreceptor degeneration in rats (Boesze-Battaglia et al., 1994). Both cholesterol and polyunsaturated docosahexaenoic acid interact with rhodopsin but have opposite effects on photocycle kinetics, further highlighting the significance of the lipid environment for receptor and cell function (Albert et al., 1996; Mitchell et al., 2001; Niu et al., 2002; Soubias and Gawrisch, 2005; Grossfield et al., 2006). Membrane cholesterol can also modulate ligand affinity, G protein coupling, and receptor oligomerization in select GPCRs, and membrane docosahexaenoic acid content was suggested to alter receptor oligomerization kinetics (Pucadyil and Chattopadhyay, 2004; Gahbauer and Böckmann, 2016). Cholesterol and endogenous ciliary oxysterols also bind to Smoothened and activate the Hh pathway (Luchetti et al., 2016; Raleigh et al., 2018). Cholesterol accessibility (or chemical activity) is further modulated by sphingolipids, which sequester cholesterol in complexes thereby blocking Hh transduction (Kinnebrew et al., 2019). Besides Hh, sphingolipids regulate several other cilia-based pathways including GPCRs (reviewed in Kaiser et al., 2020). More studies are needed to further evaluate the effects of lipid dynamics on ciliary signaling.

## Conclusion and Future Perspectives

Lipids have recently emerged as critical regulators of cilia function. The distinct lipid composition and compartmentalization of the ciliary membrane are essential for ciliary protein trafficking and transduction of cilia-based signaling cascades. The importance of lipids in cilia biology is further underscored by the fact that many ciliopathies display defects in the membrane lipid organization. Despite the key importance of lipids in cilia biology, our knowledge about cell-specific differences in the ciliary lipid composition, dynamics, and organization in distinct microdomains remains fragmented, as does our understanding of the roles that lipids play in cilia signaling. To bridge this gap in our understanding of cilia biology, systematic analysis of the lipid composition and lipid structure of sub-ciliary compartments in different cellular contexts *in vivo* is necessary. Ciliary membranes across and within organisms exhibit remarkably diverse morphologies, which are important for cell-specific cilia functions and can be modulated in response to signaling. It will be important to examine whether cell-specific differences in the ciliary lipid composition and/or dynamics also contribute to the morphological diversity of ciliary membranes. Single molecule tracking of specific lipids and mass spectrometry imaging may provide some insight into these outstanding questions and advance our understanding of the repertoire of lipid-mediated physiological functions.

## Author Contributions

The author wrote the manuscript and generated the figures.

## Conflict of Interest

The authors declare that the research was conducted in the absence of any commercial or financial relationships that could be construed as a potential conflict of interest.
